# Association between self-reported oral habits and oral health related quality of life of adolescents in Ibadan, Nigeria

**DOI:** 10.1371/journal.pgph.0003218

**Published:** 2024-05-23

**Authors:** Folake Barakat Lawal, Ejiro Idiga, Omotayo Francis Fagbule, Iyanuoluwa Jesupemi Ajayi, Folakemi Amusa, Ooreoluwa Adejumo, Mary Ebelechukwu Osuh, Orighoye Tosan Temisanren, Taiwo Akeem Lawal

**Affiliations:** 1 Department of Periodontology and Community Dentistry, University of Ibadan, Ibadan, Oyo State, Nigeria; 2 Department of Periodontology and Community Dentistry, University College Hospital, Ibadan, Oyo State, Nigeria; 3 Fellow, Consortium for Advanced Research Training in Africa (CARTA), APHRC, Nairobi, Kenya; 4 Department of Child Oral Health, University of Ibadan and University College Hospital, Ibadan, Oyo State, Nigeria; 5 Department of Surgery, Division of Pediatric Surgery, University of Ibadan and University College Hospital, Ibadan, Oyo State, Nigeria; Menzies School of Health Research: Charles Darwin University, AUSTRALIA

## Abstract

Oral habits such as nail biting, thumb/digit sucking and teeth grinding could be harmful, and may lead to teeth misalignment, anterior open bite, protruded or flared upper anterior teeth especially if they persist into adolescence. Such orofacial dysfunction may result to impairment of the Oral Health Related Quality of Life (OHRQoL) of an individual. The extent to which oral habits affect the major domains of the self-reported outcomes remains understudied especially during adolescence, a unique period of growth, where there is increased aesthetic desire, increased self-awareness, and unique social and psychological needs. The aim of this study, therefore, was to determine the prevalence of oral habits and its association with oral health related quality of life of adolescents. This cross-sectional study was conducted among 700 adolescents aged 10 to 19 years (with mean age 14.6 (±1.3) years) attending 14 secondary schools in Ibadan, Nigeria. Data were collected using a self-administered questionnaire, which assessed sociodemographic characteristics of the students, oral habits and OHRQoL with Oral Health Impact Profile 5 (OHIP-5). Data were analyzed with SPSS and p value was at <5%. Mann Whitney U statistics was used to test for associations between OHIP-5 scores and presence or absence of oral habits. Logistic regression was used for multivariate analysis. A total of 363 (51.9%; 95%CI = 48.1%–55.6%) bite their nails, 216 (30.9%; 95%CI = 27.5%–34.4%) breathe with their mouth, 122 (17.4%; 95%CI = 14.7%–20.4%) suck their lips, 89 (12.7%; 95%CI = 10.3%–15.4%) grind their teeth together and 32 (4.6%; 95%CI = 3.1%–6.7%) sucked their thumbs. A total of 403 (81.1%) adolescents who engaged in at least an oral habit reported an impairment of their OHRQoL. Painful aching in the mouth was the most frequently affected OHRQoL item reported by the adolescents who engaged in oral habits. Those who sucked their thumbs (OR = 2.3, 95%CI = 1.1–4.7, p = 0.028) and those who sucked their lips were more likely to have poorer OHRQoL (OR = 1.6, 95%CI = 1.1–2.5, p = 0.024). Oral habits were prevalent among the adolescents and affected their OHRQoL. Those who sucked their thumbs and lips were more likely to report poorer OHRQoL than those who did not.

## Introduction

Oral habits are considered as behaviors, which are repeatedly carried out in the oral cavity involving orofacial muscle actions with many of such habits having no useful benefit and could be harmful [1,2]. These habits are significant oral health problems with a high burden globally. Common oral habits include nail biting, mouth breathing, thumb/digit sucking, lip sucking, tongue thrusting, and teeth grinding [2]. Most of these habits, usually commence during infancy and are considered normal at this period because they are regarded as part of normal developmental milestone before eruption of the permanent teeth [3,4]. However, oral habits portend a risk to the teeth position, occlusion and jaws in the course of the child’s oro-facial development if the habits persist into later years [3,4]. This is because the deleterious effects of the oral habits on the oro-facial region become worse and more difficult to treat because of skeletal involvement as the habits become better established. Some of the orofacial dysfunction associated with oral habits include teeth misalignment, anterior open bite, protruded and flared upper anterior teeth among others [4,5]. Hence, consequences of oral habits have resulted into the teasing of children and adolescents, especially at school, due to their dental appearance [6–8]. In the school environment, the dental appearance of children and adolescents engaged in the oral habits has been a subject of mean-spirited and degrading teases among peers [9]. In the extreme of cases, victims of such teases are known to be distressed, suffered a negative effect on their academic performance, and have dropped out of school [9–11]. Furthermore, orofacial dysfunction resulting from such oral habits may result to impairment of the Oral Health Related Quality of Life (OHRQoL), which can be defined as a subjective assessment by an individual about how oral health affects his/her daily activities/performances [12,13]. Although some studies have reported the negative effects of oral habits on the OHRQoL [14], many of the studies did not show the extent of how oral habits affected the major domains of the self-reported outcomes. This becomes important to be able to describe the effects of oral habits on the quality of life of adolescents especially among full age range for adolescents, more so, previous studies did not capture the age range. In addition, adolescence is a unique age where there is increased aesthetic desire, increased self-awareness and unique social and psychological needs amongst other changes and, as such, important targets for oral health promotion especially in schools where many of them can be found. To develop a robust oral health promotion program targeted at adolescents, which is the aim of the upcoming school oral health promotion program in Nigeria, it becomes pertinent to provide baseline data on oral habits, an important cause of malocclusion among adolescents and describe how each oral habit affects their OHRQoL. The aim of this study, therefore, was to determine the prevalence of oral habits and its association with the oral health related quality of life of adolescents. It was therefore hypothesized that engagement in oral habits by the participants will be associated with poorer OHRQoL.

## Materials and methods

This cross-sectional study was conducted among 700 adolescents attending public secondary schools in Ibadan to assess the association between oral habits and OHRQoL. The sample size for the study was calculated using STATA at a power of 80% and estimate effect of 63% (recorded during the pilot survey) to obtain a sample size of 686, which was adjusted for a non-response rate of 2% to give a sample size of 700.

After obtaining ethical approval, permissions to conduct the study was obtained from the Ministry of Education and from principals of the selected schools and appointments were booked at the schools. Students recruited for the study were from 14 randomly selected schools from two Local Government Areas (LGAs) in Ibadan. A multistage sampling technique was utilized in selecting participants and this involved random selection of two LGAs from the 11 LGAs in Ibadan at the first stage of sampling. The second stage of sampling involved selection of seven schools from each LGA selected for the study using systematic sampling technique, making a total of 14 schools. The third stage involved selection of 50 students who met the eligibility criteria in each school using simple random sampling technique. This study is part of a larger study on the feasibility of video-supplemented training of peers and teachers at improving oral health of adolescents in Ibadan, Nigeria. The index study was conducted between 24 October 2022 and 9 December 2022.

In each school, the students were gathered together, and the details of the study was explained to them. Only students who gave assents to participate in the study, returned signed informed consent from parents/guardians and did not have a communication barrier were included in the study. Students who were ill were excluded from the study. Data for the study was obtained using a self-administered questionnaire that comprised of sociodemographic characteristics; age, gender, parent occupation, which was further classified into skilled, unskilled and dependants based on the modification of the Office of Population Censuses and Surveys tool [15,16]. Also included in the questionnaire were the self-reported oral habits and the OHIP-5 measure, which was used to assess the OHRQoL of the participants. The OHRQoL questions preceded the questions on oral habits in the questionnaire. The OHIP-5 is a short form of the Oral Health Impact Profile 14, which has worldwide acceptability in assessing OHRQoL [17]. The OHIP-5 was used because it is concise, short, easy to administer and the questions and domains have been reported to be valid for use in this environment [18–20]. It has been recommended for use in our setting [21]. The questionnaire was pretested among a total of 20 students in a school that was not selected for the main study. The students filled the questionnaire seated in the classrooms and a research assistant was there to guide them if they had questions or difficulty in filling the questionnaire. Also, the research assistants looked through the questionnaires to ensure that the questions were responded to unless participants were not willing to fill a response to the question.

The OHIP-5 score was calculated by adding the scores, which each respondent ticked as a response to the question thus, the score for each person ranged from 0 to 20 and higher OHIP-5 scores indicated poorer/worse OHRQoL.

Data obtained from the study participants was entered into SPSS for analysis. Random double data entry for 75% of the cases was done to validate data entry into SPSS. Discrepancies noted were sorted out by checking the raw data (questionnaires). The Cronbach alpha was used to assess the internal consistency of the OHIP-5 questionnaire. Categorical data were analyzed using frequencies and proportions. Continuous data such as age and total OHIP-5 scores was summarized by means and standard deviation. The OHIP-5 scores were subjected to normality test using Shapiro Wilk normality test [22]. Mann Whitney U statistics was used to test for associations between OHIP-5 scores and presence or absence of oral habits. Chi square statistics was used to assess the association between participants’ sociodemographic characteristics and impact level “higher impact” or “lower impact” on OHRQoL at bivariate analysis. For the purpose of bivariate analysis, the OHIP-5 total score was dichotomized around the mean score to yield “lower impact” for scores lower than the mean and “higher impact” for scores higher than the mean score.

Logistic regression was used for multivariate analysis for variables that were statistically significant at bivariate analysis. The level of statistical significance was set at p < 5%.

### Ethics statement

Ethical approval for the conduct of the study was obtained from the Joint University of Ibadan/University College Hospital Ethics Review Committee (UI/EC/22/0298). Permission was also obtained from the Ministry of Education in Oyo State and from the Principal of each school. A written consent was obtained from the parents of each student as mandated by the Ministry of Education, regardless of the age of the students. In addition, assent was obtained from the students (and consent from older students) before recruitment into the study. The participants were informed of their right to withdraw from the study at any point in time.

## Results

Seven hundred students were approached and all agreed to participate in the study making a response rate of 100%. The mean (SD) age was 14.6 (±1.3) years, 348 (49.7%) were males and 570 (61.4%) belonged to parents whose occupational class fell under unskilled workers (Table 1).

**Table 1:** Sociodemographic characteristics and oral habits of the adolescents.

Characteristics	n (%, 95%CI)
**Gender**	
Male	348 (49.7)
Female	352 (50.3)
**Parent occupational class**	
Skilled	99 (14.1)
Unskilled	570 (61.4)
Dependant	31 (4.4)
**Oral habit**	
Nail biting	363 (51.9, 95%CI = 48.1%–55.6%)
Mouth breathing	216 (30.9, 95%CI = 27.5%–34.4%)
Lip sucking	122 (17.4, 95%CI = 14.7%–20.4%)
Teeth grinding	89 (12.7, 95%CI = 10.3%–15.4%)
Thumb sucking	32 (4.6, 95%CI = 3.1%–6.7%)

CI: Confidence Interval.

The participants engaged in multiple oral habits. A total of 363 (51.9%) bite their nails, 216 (30.9%) breathe with their mouth, 122 (17.4%) sucked their lips, 89 (12.7%) engaged in teeth grinding and 32 (4.6%) sucked their thumbs (Table 1). A total of 497 (71.0%, 95%CI = 67.5%–74.3%) engaged in at least one of the oral habits mentioned above. There was no significant difference between age, gender or parent’s occupational class and engagement of the adolescents in any of the oral habits. There was no difference in the mean ages of those who engaged in an oral habit (14.5±1.35) years and those who did not (14.6±1.28) years, t = −0.654, p = 0.513. A comparable proportion of female adolescents (73.9%) and males (68.2%) engaged in a oral habit (X^2^ = 2.820, p = 0.093.) Students whose parents were skilled worker (74.7%), unskilled workers (70.4%) or dependants (71.0%) engaged in a habit, X^2^ = 0.792, p = 0.673.

**Fig 1:**
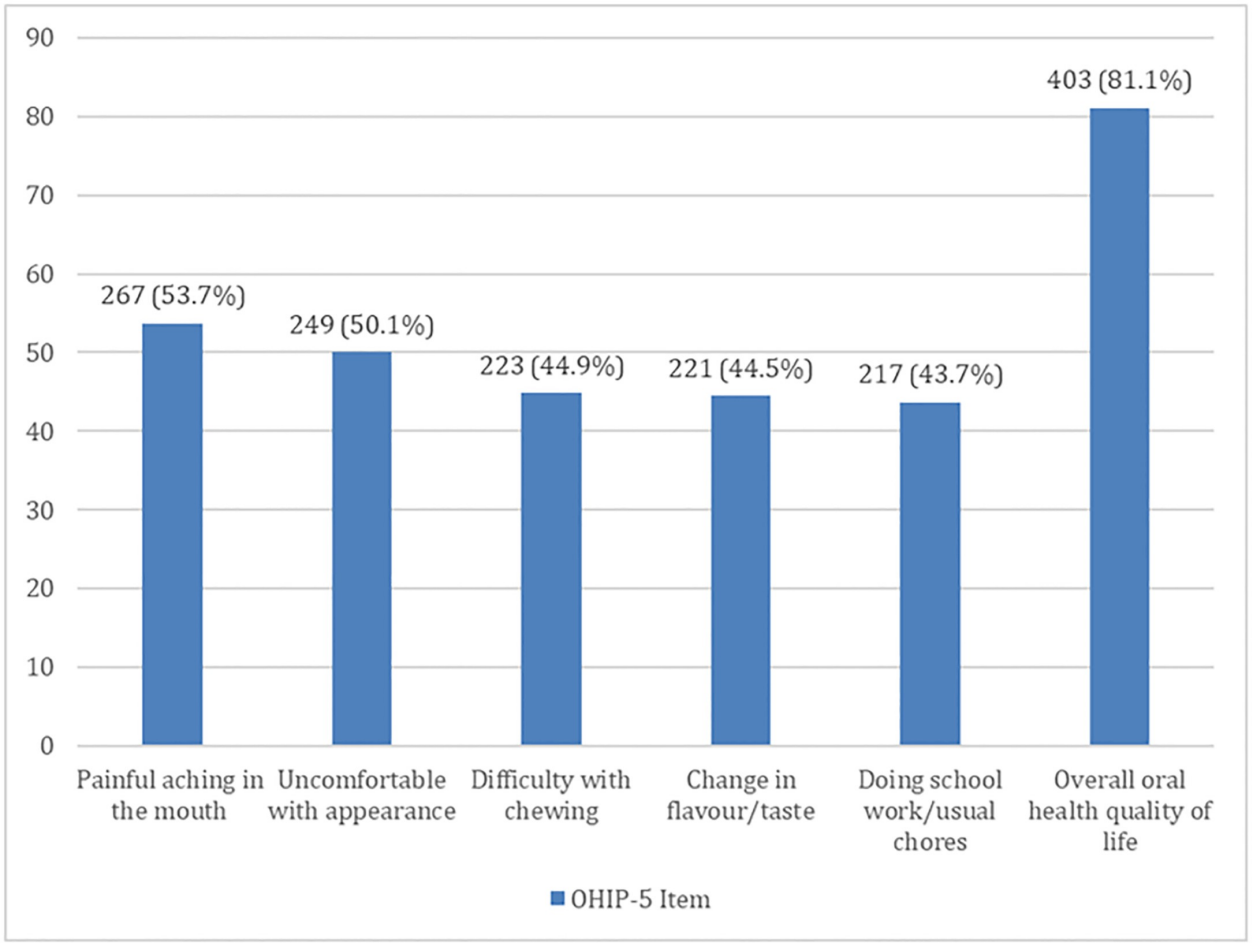
Domains of the OHIP-5 affected among participants who engaged in oral habits.

The Cronbach alpha score for the OHIP-5 questionnaire was 0.7. The OHRQoL scores of the adolescents ranged from 0–20 and the mean score was 5.3 (SD = 4.4). A total of 403 (81.1%) adolescents who engaged in at least an oral habit reported negative OHRQoL. Painful aching was the most reported OHRQoL item 267 (53.7%) among the adolescents who engaged in the oral habits (Fig 1).

Adolescents who engaged in mouth breathing reported higher effects on doing schoolwork/usual house chores (mean ranks 372.6 vs. 347.3, U = 46966.5, Z = -2.410, p = 0.016). Whereas among those who sucked their thumbs, painful aching in the mouth (mean ranks 416.9 vs. 347.3, U = 8563.0, Z = -2.019, p = 0.043), being uncomfortable about the appearance of their teeth and mouth (mean ranks 421.7 vs. 347.1, U = 8411.0, Z = -2.196, p = 0.028) as well as feeling that there had been less flavor in their food or change in taste of food (mean ranks 417.5 vs. 347.3, U = 8543.5, Z = -2.118, p = 0.034) were the OHRQoL items that were significantly affected. For those who engaged in lip sucking, difficulty with chewing food (mean ranks 386.5 vs. 342.9, U = 30866.0, Z = -2.397, p = 0.017) was the OHIP-5 item that was significantly reported. Among those who engaged in grinding of their teeth together, painful aching in their mouth was significantly reported (mean ranks 393.0 vs. 344.3, U = 23405.0, Z = -2.255, p = 0.024) (Table 2).

**Table 2:** Association between oral habits and the components of the OHIP-5 OHRQoL measure.

OHIP-5 Item	Oral habits									
	Nail biting		Mouth breathing		Thumb sucking		Lip sucking		Teeth grinding	
	Yes	No	Yes	No	Yes	No	Yes	No	Yes	No
	363	337	216	484	32	668	122	578	89	611
**Difficulty chewing**										
Mean ranks	359.1	341.3	361.8	345.4	392.2	348.5	386.5	342.9	342.5	351.7
Median score	0.0	0.0	0.0	0.0	1.0	0.0	1.0	0.0	0.0	0.0
Mann Whitney U	58054.5		49824.0		9353.0		30866.0		26479.5	
Z	-1.289		-1.097		-1.323		-2.397		-0.441	
P value	0.197		0.273		0.186		0.017*		0.659	
**Painful aching**										
Mean ranks	363.8	336.2	333.2	358.2	416.9	347.3	377.2	344.9	393.0	344.3
Median score	1.0	1.0	0.0	1.0	2.0	1.0	1.0	1.0	1.0	1.0
Mann Whitney U	56343.5		48536.0		8563.0		31996.0		23405.0	
Z	-1.915		-1.605		-2.019		-1.707		-2.255	
P value	0.055		0.108		0.043*		0.088		0.024*	
**Uncomfortable with appearance of teeth**										
Mean ranks	354.8	345.9	352.4	349.7	421.7	347.1	365.8	347.3	370.7	347.6
Median score	0.0	0.0	0.0	0.0	1.5	0.0	1.0	0.0	1.0	0.0
Mann Whitney U	59600.5		51871.0		8411.0		33387.5		25390.0	
Z	-0.631		-0.175		-2.196		-0.993		-1.088	
P value	0.528		0.861		0.028*		0.321		0.276	
**Less flavor in food/change in taste**										
Mean ranks	349.0	352.1	354.8	348.6	417.5	347.3	355.1	349.5	382.6	345.8
Median score	0.0	0.0	0.0	0.0	1.5	0.0	0.0	0.0	1.0	0.0
Mann Whitney U	60623.5		51349.5		8543.5		34701.0		24337.0	
Z	-0.224		-0.412		-2.118		-0.303		-1.766	
P value	0.823		0.680		0.034*		0.762		0.077	
**Difficulty doing usual jobs/schoolwork**										
Mean ranks	354.6	346.1	372.6	347.3	385.6	348.8	359.0	348.7	372.6	347.3
Median score	0.0	0.0	0.0	0.0	0.5	0.0	0.0	0.0	0.0	0.0
Mann Whitney U	59671.0		46966.5		9565.5		34227.0		25912.0	
Z	-0.628		-2.410		-1.128		-0.570		-0.805	
P value	0.530		0.016*		0.260		0.569		0.421	

* Statistically significant.

A higher proportion of adolescents who sucked their thumb 13 (40.6%) had poorer OHRQoL compared with those 155 (23.2%) who did not suck their thumb (X^2^ = 5.081, p = 0.024).

Also, a higher proportion of adolescents who sucked their lips reported poorer OHRQoL (32.0% vs. 22.3%, X^2^ = 5.142, p = 0.023) (Table 3).

**Table 3:** Association between oral habits and OHRQoL.

Oral habit	OHRQoL		X^2^	p value
	OHIP<5	OHIP≥5		
**Nail biting**				
Present	268 (73.8)	95 (26.2)	1.948	0.163
Absent	264 (78.3)	73 (21.7)		
**Mouth breathing**				
Present	158 (73.1)	58 (26.9)	1.393	0.238
Absent	374 (77.3)	110 (22.7)		
**Lip sucking**				
Present	83 (68.0)	39 (32.0)	5.142	0.023*
Absent	449 (77.7)	129 (22.3)		
**Teeth grinding**				
Present	65 (73.0)	24 (27.0)	0.492	0.483
Absent	467 (76.4)	144 (23.6)		
**Thumb sucking**				
Present	19 (59.4)	13 (40.6)	5.081	0.024*
Absent	513 (76.8)	155 (23.2)		

* Statistically significant.

Multivariate analysis showed that those who sucked their thumbs (OR = 2.3, 95%CI = 1.1–4.7, p = 0.028) and those who sucked their lips were more likely to have poorer OHRQoL (OR = 1.6, 95%CI = 1.1–2.5, p = 0.024) (Table 4).

**Table 4:** Multivariate analysis of the association between oral habits and OHRQoL.

Oral habit	OR	95%CI	p value
**Thumb sucking**			
Yes	2.3	1.1–4.7	0.028*
**Lip sucking**			
Yes	1.6	1.1–2.5	0.024*

* Statistically significant.

## Discussion

This study examined the prevalence of oral habits among adolescents aged 10 to 19 years attending secondary schools in Ibadan, Nigeria, and the association of those habits with their OHRQoL. The study found that more than two-thirds of the participants engaged in at least one oral habit and those who engaged in the oral habits were more likely to have poorer OHRQoL. Pain was the most reported domain of OHRQoL reported by those with oral habits.

A few (4.6%) of the participants in this study engaged in thumb-sucking. The prevalence of thumb sucking in this study is similar to the 4.2% that was reported in a previous study conducted in Lagos, Nigeria [23]. In contrast, other studies within Nigeria, [24–26] and outside Nigeria [2,27–30] reported a higher prevalence ranging from 6.2% to 25.2%. Unlike the index study, all the studies that reported a higher prevalence had much younger participants, with some including one-year-old babies, which is understandable as digit sucking is a habit that is commoner among babies and small children [3,4]. Lower prevalence (2.3%) than that observed in this study was reported among 12 and 15-year-old school-going adolescents in Trinidad [28].

This study demonstrated an association between thumb sucking and OHRQoL. Specifically, a higher proportion of those who engaged in thumb-sucking compared to those who did not, complained about painful aching in their mouth, were uncomfortable with the appearance of their teeth and perceived less flavor in food/change of taste. Thumb sucking is a major cause of malocclusion [4], which increases the risk of poor oral hygiene and oral health problems such as dental caries and gum diseases that are the common sources of pain in the mouth. Similarly, thumb sucking distorts proper teeth arrangement, which causes poor aesthetics of the dentition, leading to displeasure, a reason why the adolescents reported being uncomfortable with the appearance of their teeth. Children who engaged in digit sucking may perceive less flavor in food or feel a change of taste in food due to a phenomenon called “taste adaptation”, which occurs as a result of the constant exposure of their taste buds to saliva. This can lead to a desensitization of the taste buds, reducing their ability to perceive certain flavors and tastes [31]. Furthermore, the constant presence of saliva in the mouth can change the composition of the oral microbiota, which can also affect the perception of taste [32]. Studies have shown that changes in the oral microbiota can lead to altered taste perception, as certain bacteria may produce compounds that interact with taste receptors [33,34]. It is noteworthy that the effects of digit sucking on taste perception are typically temporary and may improve once the habit is stopped [32]. Overall, this study showed that thumb sucking was a predictor of poor OHRQoL among adolescents.

The prevalence of lip-sucking in this study is high, with almost one out of every five participants engaged in this oral habit. This prevalence is similar to the prevalence of lip sucking reported among 4–18-year-old school children in Yenagoa, Southern Nigeria where a prevalence of 19.1% was reported [25]. However, lower prevalence was reported in Southwestern Nigeria (6.3% and 1.4%.) among 5–12-year-olds in Lagos [23], and among 1–12-year-olds in Ile-Ife [26], respectively. Studies outside Nigeria also reported widely different prevalence: 4.0% in the USA [30], 4.9% [2] and, 6.3% in India [35], 9.1% in Trinidad [28], 29.4% in Egypt [29], and 31.3% in Brazil [27]. The differences in the studies may be attributed to the ages and composition of the study participants.

Lip sucking was also a predictor of poor OHRQoL in this study. Participants who engaged in lip-sucking were more likely to complain about the difficulty in chewing compared to those who did not engage in the habit. Lip sucking can interfere with the normal development of the muscles used for chewing and swallowing, leading to difficulties with these functions [4]. Over time, this can lead to an abnormal alignment of the teeth that can affect chewing ability [4], which can also lead to malocclusion and changes in the position of the teeth, thus, inefficient or ineffective chewing.

Mouth breathing was also assessed among the study participants and about one-third of them engaged in the habit. There is a dearth of literature from Nigeria that assessed mouth breathing as one of the oral habits. However, previous studies outside Nigeria have reported a wide range of prevalence including; 36.1% in Turkey [36], 23.3% in USA [30], 15.9% in Egypt [29], and 5.7% and 4.3% in India [2,35].

The study showed that those who engaged in mouth breathing reported having “difficulty doing their normal job/school work” compared to others. This finding is likely because mouth breathers are deprived of good quality sleep since they find it difficult to breathe while asleep and with their mouth closed. Due to the lack of good quality sleep, they are likely to feel tired and lethargic during the day, thus, affecting their daily activities and school.

Teeth grinding, also called bruxism, was observed in 12.7% of the adolescents, similar to the prevalence of 15.0% reported in Yenogoa, Nigeria [25]. The prevalence is however, higher than the reports of 0.92% among 5-12-year-olds in Lagos [23] and 1.4% among 1-12-year-olds in Ile-Ife [26]. Wide prevalence had been reported from studies conducted outside Nigeria, ranging from 2.0% to 17.7% [2,27,35]. As opined by Manfredini *et al*. [37] following a systematic review of the literature, the wide differences from the various studies may be because of the different age groups and inclusion of self-reports in some studies [37]. It has also been noted that it may be difficult to get an accurate estimate of the prevalence of bruxism because many of those who engage in the habit, especially during sleep, may be unaware of it [38].

Bruxism often leads to problems such as tooth wears, fractures, and pain in the oro-facial area [38]. These adverse effects negatively affect the OHRQoL of many who engage in the habit [39]. Thus, it is understandable that more of those who engaged in teeth grinding in this study reported “painful aching” compared to those who did not engage in the habit.

Similar to the present study, previous studies reported nail-biting as the most common oral habit among their study participants, ranging from 12.1% to 65.5% [23,25,27–29,35,40]. Nail biting is the only oral habit in this study without a significant effect on any of the domains of the OHIP-5. Although nail biting has the potential to cause oral health problems including the misalignment of teeth, the habit has to be persistent and prolonged to cause these problems [41] and may not be the case in this study.

Overall, seven out of every 10 participants in this study engaged in at least one oral habit. The proportion of those with at least one habit from previous studies within and outside the country also differs widely, ranging from 13.1%–86.5% [2,23–30,35]. Other than the differences in the age-range of the study participants, other reasons for the wide differences may have to do with the data collection method, making it difficult to compare the results. For example, some of the studies used an objective assessment of the oral habits by examining the participants’ digits and nails for digit sucking and nail biting and examining their mouth and oral musculature for other habits including tongue thrusting, lip sucking, and teeth grinding [26,29]. However, other studies only based their assessments on self-reports [23,35]. Similarly, while some studies reported the current practice of oral habits, others assessed both the current and past history [28]. In reporting the prevalence of individual habits, some studies made the total population the denominator [2,30]. while others made the number of those engaged in at least one habit the denominator [23,26]. These made it difficult to compare the results and may lead to wrong interpretations. Hence, we suggest that there should be a consensus on the assessment and reportage of these habits. However, an important strength of this study is the use of the OHIP-5, a five item, four dimensional OHRQoL tool, which is less cumbersome for use especially among adolescents in schools. The evolvement of the OHIP tool from the 49-item tool down to the 5-item tool with its validity and importance of its ease of use [18–20,42,43] has gained global recognition.

The prevalence of oral habits observed in this study is very high and needs to be promptly addressed considering that these habits are potential risks for adverse effects including malocclusion, developmental and functional defects of the oro-facial region, with negative effects on OHRQoL. It, therefore, becomes pertinent to institute appropriate interventions that will involve children/adolescents, parents, teachers, healthcare professionals, government, and other stakeholders to prevent the initiation of these habits and assist those who have commenced the habits to discontinue [25,44–47].

School teachers can also play a role in preventing the habits among their pupils/students by counseling them and their parents. However, studies have also reported that teachers also lacked adequate knowledge and had unsatisfactory attitudes about oral habits [48]. Hence, it is important that health professionals, especially dentists, educate the populace and teachers about issues around oral habits and how to avoid/correct them. The education should not be limited to the populace alone, even physicians/pediatricians should be educated about the peculiarities of oral habits, as some may not have the requisite knowledge about the habits and the need to refer patients with oral habits for expert review and management [49].

This study is not without its limitations, being a cross-sectional design, it cannot assess causality. However, this is not a problem because the authors were not set out to assess causality, but to assess for association between oral habits and OHRQoL. In addition, the study is prone to self-report bias because the assessment of oral habits was based on self-report. Although to minimize this potential bias, the questionnaires were anonymized and the participants were assured that their responses would be kept confidential. The questions on oral habits were developed from extensive review of the literature and as such oral habits not commonly reported in the literature may have been omitted from the questions. It is worthy of note that children who had communication barrier or were ill at the time of the study were excluded from the present study. It is uncertain if these children had different oral health habits and impairment on OHRQoL compared to those included in the study, so caution should be exercised in the generalizability of the research findings to these group of adolescents. However, the purpose of the study was to be able to describe the prevalence of oral habits and its association with OHRQoL among adolescents to provide baseline data needed for planning appropriate intervention among them.

In conclusion, this study showed that the prevalence of engaging in at least one oral habit was very high and nail-biting was the most common oral habit. Thumb sucking, lip sucking, teeth grinding, and mouth breathing all had significant association with at least one of the domains of the OHIP-5 resulting in poorer OHRQoL. Both digit sucking and lip sucking/tongue thrusting habits were found to be predictors of poor OHRQoL among the participants.
